# Clinical features and outcomes of combined hepatocellular carcinoma and cholangiocarcinoma versus hepatocellular carcinoma versus cholangiocarcinoma after surgical resection: a propensity score matching analysis

**DOI:** 10.1186/s12876-020-01586-4

**Published:** 2021-01-07

**Authors:** Chih-Wen Lin, Tsung-Chin Wu, Hung-Yu Lin, Chao-Ming Hung, Pei-Min Hsieh, Jen-Hao Yeh, Pojen Hsiao, Ya-Ling Huang, Yu-Chan Li, Ya-Chin Wang, Chih-Wen Shu, Yaw-Sen Chen

**Affiliations:** 1 Division of Gastroenterology and Hepatology, E-Da Dachang Hospital, I-Shou University, Kaohsiung, Taiwan; 2 Division of Gastroenterology and Hepatology, Department of Medicine, E-Da Hospital, I-Shou University, Kaohsiung, Taiwan; 3 Division of General Surgery, Department of Surgery, E-Da Hospital, and School of Medicine, College of Medicine, I-Shou University, No. 1, Yida Road, Jiaosu Village, Yanchao District, Kaohsiung City, 82445 Taiwan; 4 Department of Surgery, E-Da Cancer Hospital, I-Shou University, Kaohsiung, Taiwan; 5 School of Medicine, College of Medicine, I-Shou University, Kaohsiung, Taiwan; 6 School of Chinese Medicine, College of Chinese Medicine, and Research Center for Traditional Chinese Medicine, China Medical University, Taichung, Taiwan

**Keywords:** Combined hepatocellular carcinoma and cholangiocarcinoma, Overall survival, Recurrence, Prognosis

## Abstract

**Background:**

Combined hepatocellular carcinoma and cholangiocarcinoma (cHCC-CC) is an infrequent type of primary liver cancer that comprises hepatocellular carcinoma (HCC) and cholangiocarcinoma (CC). This study investigated the clinicopathological features and prognosis among cHCC-CC, HCC, and CC groups.

**Methods:**

We prospectively collected the data of 608 patients who underwent surgical resection for liver cancer between 2011 and 2018 at E-Da Hospital, I-Shou University, Kaohsiung, Taiwan. Overall, 505 patients with cHCC-CC, HCC, and CC were included, and their clinicopathological features, overall survival (OS), and recurrence were recorded. OS and recurrence rates were analyzed using the Kaplan–Meier analysis.

**Results:**

In the entire cohort, the median age was 61 years and 80% were men. Thirty-five (7.0%) had cHCC-CC, 419 (82.9%) had HCC, and 51 (10.1%) had CC. The clinicopathological features of the cHCC-CC group were more identical to those of the HCC group than the CC group. OS was significantly lower in the cHCC-CC group than in the HCC group but was not significantly higher in the cHCC-CC group than in the CC group. The median OS of cHCC-CC, HCC, and CC groups was 50.1 months [95% confidence interval (CI): 38.7–61.2], 62.3 months (CI: 42.1–72.9), and 36.2 months (CI: 15.4–56.5), respectively. Cumulative OS rates at 1, 3, and 5 years in cHCC-CC, HCC, and CC groups were 88.5%, 62.2%, and 44.0%; 91.2%, 76.1%, and 68.0%; and 72.0%, 48.1%, and 34.5%, respectively. After propensity score matching (PSM), OS in the cHCC-CC group was not significantly different from that in the HCC or CC group. However, OS was significantly higher in the HCC group than in the CC group before and after PSM. Furthermore, the disease-free survival was not significantly different among cHCC-CC, HCC, and CC groups before and after PSM.

**Conclusion:**

The clinicopathological features of the cHCC-CC group were more identical to those of the HCC group than the CC group. The OS rate was significantly lower in the cHCC-CC group than the HCC group. However, after PSM, OS and disease-free survival in the cHCC-CC group were not significantly different from those in the HCC or CC group.

## Background

Combined hepatocellular carcinoma and cholangiocarcinoma (cHCC-CC) is a rare type of primary liver cancer (PLC) [1, 2], and its incidence accounts for 0.4–14.2% of PLC [1–5]. In 1949, Allen and Lisa defined cHCC-CC as the intermingling of both HCC and CC components and classified cHCC-CC into the three types [4].

An accurate preoperative diagnosis of cHCC is challenging [6], and most cases are confirmed based on postoperative histopathology. Notably, the clinicopathological features of cHCC-CC were more different to those of CC compared with HCC [2, 5, 7–12]. By contrast, cHCC-CC was genetically identical to CC compared with HCC in a molecular study [2, 5, 7, 13–21]. In addition, the 5-year overall survival (OS) rate of patients with cHCC-CC were very different between 0 and 50% [7, 22–24], which is poorer or similar compared with that of patients with HCC [5, 7, 12, 15–21, 25]. The recurrence pattern of cHCC-CC was different from that of HCC compared with CC [26]. However, the demographics, pathological features, and prognosis of cHCC-CC remain largely unknown. Hence, this study aims to investigate the clinicopathological features and clinical outcomes of patients with cHCC-CC, HCC, and CC. Furthermore, we compared clinical outcomes among patients with cHCC-CC, HCC, and CC after propensity score matching (PSM) related to sex, age, cirrhosis, Child–Pugh (CP) class, tumor size, tumor number, and American Joint Committee on Cancer (AJCC) stage.

## Methods

### Patients and follow-up

**Fig. 1:**
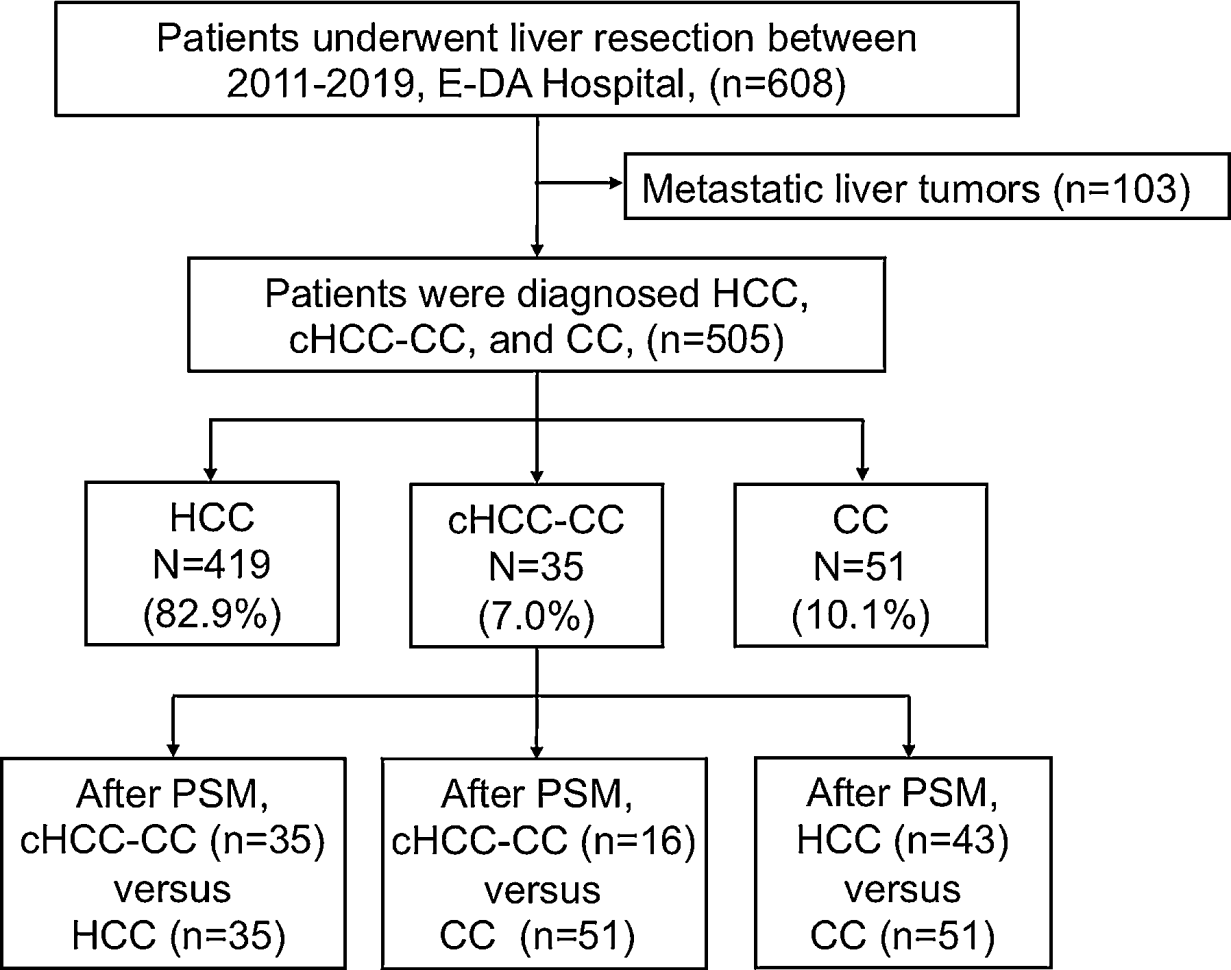
Study flowchart and inclusion of participants

This is a prospectively cohort study inclusive of 608 liver cancer patients underwent surgical resection from 2011 to 2018 at E-Da Hospital, I-Shou University, Kaohsiung, Taiwan. Of these, 103 patients were excluded because of metastatic liver tumors. Finally, our prospective study included 505 patients diagnosed with cHCC-CC, HCC, and CC confirmed by pathological findings (Fig. 1). This study was approved by the Institutional Review Board of E-Da Hospital, I-Shou University (EMRP32100N). Patients were diagnosed with cHCC-CC, HCC, and CC based on histological confirmation. Clinicopathological information, such as demographic data, etiology, liver cirrhosis, CP class, operative methods, tumor factors, alpha-fetoprotein (AFP) level, vascular invasion, metastasis, mortality, and recurrence were examined as our previous study. Liver cirrhosis was diagnosed based on pathologic findings. The liver preserved functional was evaluated using the CP scoring system [27].

Patients were followed up every 3–6 months through abdominal ultrasound, computed tomography, or magnetic resonance imaging. OS was defined as the time from the date of HCC diagnosis to the date of death, the last follow-up, or the end of the study in June 2019, whichever came first. disease-free survival (DFS) was defined as the time from the date of HCC diagnosis to the date of recurrence, the last follow-up, or the end of the study in June 2019, whichever came first.

### Data analysis and statistics

Continuous data are expressed as medians and ranges. Categorical data are described as numbers and percentages. Normally distributed continuous variables were compared using Student’s *t* test or one-way ANOVA test, and Wilcoxon rank-sum statistics were applied when two groups were compared and continuous variables were not normally distributed. The chi-squared test was used to compare categorical variables. Recurrence rates by various disease statuses were calculated and expressed per 100 person-years. Mortality rates by different disease statuses were calculated and expressed per 100 person-years. OS and DFS was evaluated using the Kaplan–Meier method. Statistical differences in OS among subgroups were examined using the log-rank test. Median OS is presented as the median and 95% confidence interval (CI).

Logistic regression was used to perform PSM by using patients’ sex, age, cirrhosis status, CP class, tumor size, tumor number, and AJCC stage to reduce bias in the analysis. Each group was matched with the control group (cHCC-CC group or CC group) according to the generated PSM by using a caliper width of 0.05. On the completion of matching, baseline covariates were compared using the paired t-test or Mann–Whitney U test for continuous variables and the chi-square test for categorical variables. A *p* value of < 0.05 was considered statistically significant. All statistical analyses were performed using SPSS version 23.0 (SPSS, Chicago, IL, USA).

## Results

### Patients’ baseline characteristics

Overall, 505 patients were included in this study (Fig. 1). The clinicopathological features of all cohorts are presented in Table 1. In the entire cohort, the median age was 61 years, the majority (80%) were men, approximately half had hepatitis B virus (HBV, 45.0%), one-fourth had hepatitis C virus (HCV, 24.6%), and 20.2% had a history of alcohol use. Approximately 32.3% of patients had liver cirrhosis, with the majority (96.8%) having CP class A. Several patients (45.2%) had tumors ≥ 5 cm in size, and 10.2% had multiple tumors.

**Table 1:** Demographic characteristics of patients with combined hepatocellular carcinoma and cholangiocarcinoma, hepatocellular carcinoma, and cholangiocarcinoma

Characteristics	Total (n = 505)	cHCC-CC (n = 35)	HCC (n = 419)	CC (n = 51)	*p* value
Male	404 (80.0)	28 (80.0)	343 (81.9)^b^	33 (64.7)	0.015
Age (years)	61 (32–87)	57 (37–79)^a^	62 (32–87)	60 (33–85)	0.211
Hypertension	208 (41.2)	16 (45.7)	178 (42.5)^b^	14 (27.5)	0.102
Diabetes Mellitus	119 (23.60	3 (8.6)^a^	102 (24.3)	14 (27.5)^c^	0.085
Smoking	208 (48.6)	27 (47.4)	87 (20.8)	8 (15.7)	0.695
Alcohol use	102 (20.2)	7 (20.0)	74 (17.7)	4 (7.8)^c^	0.043
HBV positive	227 (45.0)	15 (42.9)	186 (44.4)	26 (51.0)	0.649
HCV positive	124 (24.6)	8 (22.9)	98 (23.9)	18 (35.3)	0.171
Total Bilirubin	0.95 ± 0.42	0.93 ± 0.32	0.95 ± 0.42	0.94 ± 0.46	0.982
Albumin	4.1 ± 0.3	4.0 ± 0.3	4.1 ± 0.3	4.2 ± 0.2	0.308
INR	1.04 ± 0.07	1.03 ± 0.04	1.04 ± 0.07	1.05 ± 0.12	0.451
Cirrhosis	163 (32.3)	12 (34.4)	145 (34.6)^b^	6 (11.8)^c^	0.004
Child–Pugh class A	489 (96.8)	35 (100)	403 (96.5)	51 (100)	0.399
Edmondson–Steiner Grades, I–II	322 (63.7)	18 (51.4)	292 (69.6)^b^	12 (23.5)	0.404
Tumor size	5.3 ± 3.3	5.8 ± 2.1	5.2 ± 3.4	5.3 ± 2.4	0.623
Tumor size ≥ 5 cm	225 (45.2)	24 (68.6)^a^	178 (42.5)^b^	23 (52.3)	0.007
Tumor number (≥ 2)	51 (10.2)	3 (8.6)	46 (11.0)	2 (4.5)	0.385
AFP (ng/mL) ≥ 200	91(21.3)	8 (22.9)	115 (27.4)^b^	3 (5.9)^c^	0.003
ICG%	11.1 ± 7.9	10.7 ± 5.5	11.3 ± 8.3	9.6 ± 5.5	0.359
Operative margin > 1 cm	361 (71.4)	25 (71.4)	306 (73.0)^b^	30 (58.8)^c^	0.003
Major hepatectomy	257 (50.8)	19 (54.2)	203 (48.4)^b^	35 (68.6)^c^	0.007
Microvascular invasion	152 (30.1)	13 (37.1)	131 (31.3)^b^	8 (15.7)^c^	0.047
Macrovascular invasion	72 (14.3)	5 (14.3)	66 (15.8)^b^	1 (2.0)^c^	0.029
Lympho nodules metastasis	20 (4.0)	7 (20.2)^a^	5 (1.2)^b^	8 (15.7)	< 0.0001
Distal metastasis	10 (2.0)	0 (0)	7 (1.7)^b^	3 (5.9)	0.086
AJCC stage, I–II	383 (75.8)	27 (77.1)	320 (76.3)^b^	36 (70.5)	< 0.0001
Antiviral therapy	282 (55.8)	18 (51.4)	239 (57.0)	25 (49.0)	0.168
Recurrence	135 (26.7)	9 (25.7)	120 (28.6)^b^	6 (11.8)	0.036
Recurrence per 100 person-years	33.7	42.1	38.5^b^	25.3	< 0.0001
Mortality	149 (29.5)	14 (40.0)^a^	108 (25.8)^b^	27 (52.8)	< 0.0001
Mortality per 100 person-years	94.1	104.1^a^	86.7^b^	113.8	< 0.0001
Follow-up times (months)	38 (1–94)	31 (4–75)^a^	40 (1–94)^b^	26 (1–85)	< 0.0001

Data are presented as the median (range) or number (percentage).

*HBV* Hepatitis B virus, *HCV* Hepatitis C virus, *INR* international normalized ratio, *AFP* alpha-fetoprotein, *ICG* indocyanine green, *AJCC* American Joint Committee on Cancer

^a^*p* < 0.05, cHCC-CC versus HCC

^b^*p* < 0.05, cHCC-CC versus CC

^c^*p* < 0.05, HCC versus CC

Among the 505 patients, 35 (7.0%) patients had cHCC-CC, 419 (82.9%) had HCC, and 51 (10.1%) had CC (Table 1). Significant intergroup differences were observed regarding factors such as sex, alcohol use, tumor size ≥ 5 cm, AFP level ≥ 200 ng/mL, operative margin > 1 cm, major hepatectomy, microvascular invasion, macrovascular invasion, AJCC stage I–II, recurrence, recurrence per 100 person-years, mortality, mortality per 100 person-years, and median follow-up time.

The cHCC-CC group had the highest proportion of patients with hypertension, smoking, alcohol use, CP class A, tumor size ≥ 5 cm, microvascular invasion, lymph node metastasis, AJCC stage I–II, recurrence per 100 person-years, and mortality per 100 person-years. By contrast, the HCC group had more patients with old age, male, cirrhosis, Edmondson–Steiner grades I-II, tumor number, AFP level ≥ 200 ng/mL, ICG%, operative margin > 1 cm, macrovascular invasion, and antiviral therapy. Moreover, the CC group was noted to more likely have diabetes mellitus, HBV, HCV, CP class A, major hepatectomy, and distal metastasis, as presented in Table 1.

Significant differences were observed between cHCC-CC and HCC groups in terms of age, diabetes mellitus status, tumor size ≥ 5 cm, lymph node metastasis, mortality, mortality per 100 person-years, and median follow-up time. Furthermore, significant differences were observed between cHCC-CC and CC groups in terms of diabetes mellitus, alcohol use, cirrhosis, AFP level ≥ 200 ng/mL, operative margin > 1 cm, major hepatectomy, microvascular invasion, and macrovascular invasion. In addition, significant differences were noted between HCC and CC groups in terms of sex, hypertension, cirrhosis, Edmondson–Steiner Grades I–II, tumor size ≥ 5 cm, AFP level ≥ 200 ng/mL, operative margin > 1 cm, major hepatectomy, microvascular invasion, macrovascular invasion, lymph node metastasis, AJCC stage I–II, recurrence, recurrence per 100 person-years, mortality, mortality per 100 person-years, and median follow-up time.

### Overall survival in the entire cohort and different groups

**Fig. 2:**
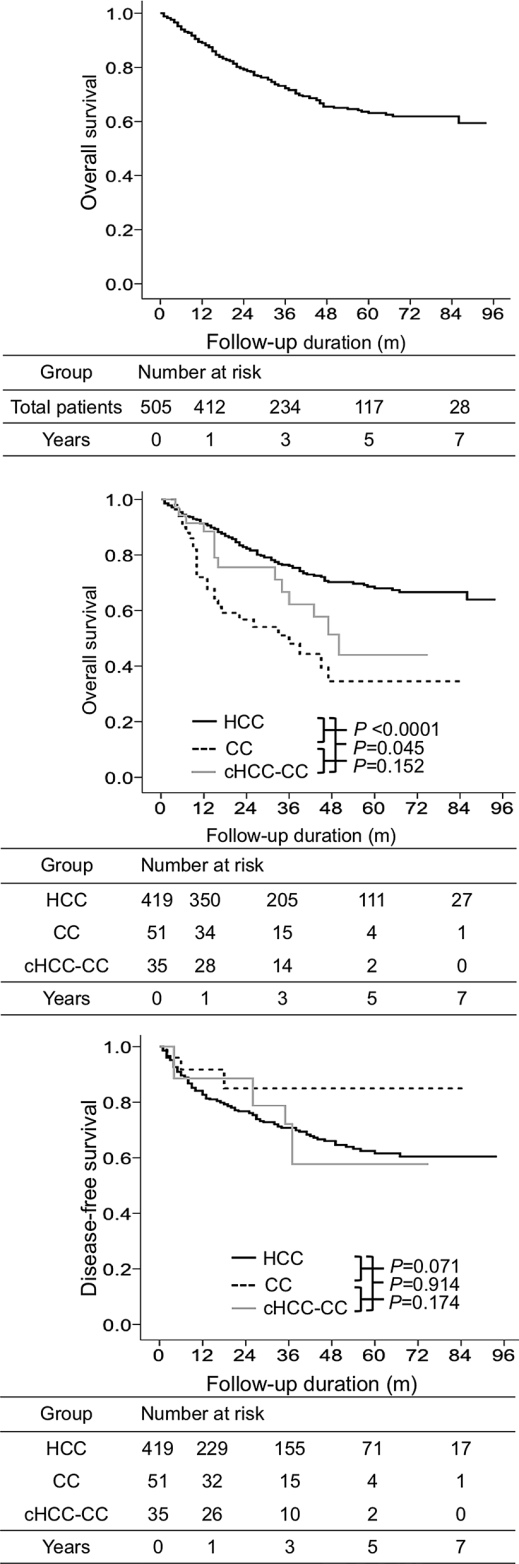
Overall survival and disease-free survival in the entire cohort, cHCC-CC, HCC, and CC groups. Overall survival in the whole cohort (**a**). Overall survival based on Cox regression analysis in cHCC-CC, HCC, and CC groups (**b**). Disease-free survival based on Cox regression analysis in cHCC-CC, HCC, and CC groups (**c**)

Of the 505 patients, 149 (29.5%) died, and the median follow-up duration was 38 months (range: 1–94 months; Table 1). The mortality rate was 94.1 per 100 person-years. Cumulative OS at 1, 3, 5, and 7 years was 89.0%, 72.2%, 63.1%, and 61.9%, respectively (Fig. 2a). OS was significantly better in the HCC group than in the cHCC-CC group [hazard ratio (HR): 1.77; 95% CI: 1.01–3.09, *p* = 0.045, Fig. 2b]. OS was significantly better in the HCC group than in the CC group (HR: 2.84; 95% CI: 1.85–4.34, *p* < 0.0001, Fig. 2b). Moreover, OS was not significantly better in the cHCC-CC group than in the CC group (HR: 1.60; 95% CI: 0.84–3.05, *p* = 0.152, Fig. 2b). The median OS for cHCC-CC, HCC, and CC groups was 50.1 months (95% CI: 38.7–61.2), 62.3 months (CI: 42.1–72.9), and 36.2 months (CI: 15.4–56.5), respectively. The mortality was 104.1, 86.7, and 113.8 per 100 person-years in cHCC-CC, HCC, and CC groups, respectively. The cumulative OS rates at 1, 3, 5, and 7 years in cHCC-CC, HCC, and CC groups were 88.5%, 62.2%, 44.0%, and 44.0%; 91.2%, 76.1%, 68.0%, and 66.6%; and 72.0%, 48.1%, 34.5%, and 34.5%, respectively (Fig. 2b).

### Recurrence in the entire cohort and different groups

Of the 505 patients, 135 (26.7%) had recurrence (Table 1). The recurrence rate was 33.7 per 100 person-years. The disease-free survival rates were not significantly different among cHCC-CC, HCC, and CC groups (all *p* > s 0.05, Fig. 2c). The median time to recurrence for cHCC-CC, HCC, and CC were 26.2 months (95% CI: 9.85–56.4), 10.9 months (CI: 7.15–12.8), and 6.8 months (CI: 1.47–10.5), respectively. The cumulative DFS rates at 1, 3, 5, and 7 years in cHCC-CC, HCC, and CC groups were 88.6%, 72.2%, 57.7%, and 57.7%; 82.7%, 70.8%, 61.6%, and 60.4%; and 91.7%, 80.9%, 75.1%, and 75.1%, respectively (Fig. 2c).

### Overall survival in different groups after PSM

PSM was performed using sex, age, cirrhosis status, CP class, tumor size, tumor number, and AJCC stage, and no significant differences were noted regarding crucial features (Tables 2, 3).

**Table 2:** Comparison of combined hepatocellular carcinoma and cholangiocarcinoma versus hepatocellular carcinoma or cholangiocarcinoma after propensity score matching

Variable	cHCC-CC (n = 35)	HCC (n = 35)	*p* value	cHCC-CC (n = 35)	CC (n = 35)	*p* value
Male	28 (80.0)	29 (82.9)	0.759	28 (80.0)	25 (71.4)	0.339
Age (years)	57 (37–79)	57 (35–81)	0.894	57 (37–79)	60 (35–80)	0.199
Smoking	7 (20.0)	13 (37.1)	0.112	7 (20.0)	4 (11.4)	0.324
Alcohol use	10 (28.6)	11 (31.4)	0.794	10 (28.6)	4 (11.4)	0.209
HBV positive	15 (42.9)	20 (57.1)	0.232	15 (42.9)	17 (48.6)	0.631
HCV positive	8 (22.9)	5 (14.3)	0.356	8 (22.9)	13 (37.1)	0.192
Total bilirubin	0.93 ± 0.32	0.85 ± 0.28	0.455	0.93 ± 0.32	0.95 ± 0.45	0.861
Albumin	4.1 ± 0.3	4.1 ± 0.3	0.481	4.1 ± 0.3	4.2 ± 0.3	0.102
INR	1.04 ± 0.04	1.06 ± 0.06	0.060	1.04 ± 0.04	1.05 ± 0.11	0.446
Cirrhosis	12 (34.3)	11 (31.4)	0.799	12 (34.3)	6 (17.1)	0.107
Child–Pugh class A	35 (100)	34 (97.1)	0.801	35 (100)	35 (100)	1.000
Edmondson–Steiner Grades, I–II	12 (34.2)	13 (37.5)	0.313	12 (34.2)	11 (31.4)	0.861
Tumor size	6.6 ± 3.5	7.4 ± 4.3	0.055	6.6 ± 3.5	6.0 ± 2.8	0.125
Tumor size ≥ 5 cm	24 (68.6)	25 (71.4)	0.794	24 (68.6)	19 (54.3)	0.220
Tumor number (≥ 2)	3 (8.6)	6 (17.1)	0.284	3 (8.6)	1 (2.9)	0.303
AFP (ng/mL) ≥ 200	8 (22.9)	13 (37.1)	0.192	8 (22.9)	3 (8.6)	0.101
ICG%	10.7 ± 5.4	9.2 ± 5.0	0.232	10.7 ± 5.4	9.3 ± 5.6	0.268
Operative margin > 1 cm	12 (34.3)	11 (31.4)	0.799	12 (34.3)	18 (51.4)	0.198
Major hepatectomy	15 (42.8)	16 (45.7)	0.783	15 (42.8)	20 (57.1)	0.328
Microvascular invasion	13 (37.5)	12 (34.2)	0.803	13 (37.5)	6 (17.1)	0.060
Macrovascular invasion	5 (14.3)	12 (34.2)	0.056	5 (14.3)	1 (1.9)	0.088
Lympho nodules metastasis	7 (20.0)	3 (8.5)	0.095	7 (20.0)	7 (20.0)	1.000
Distal metastasis	0 (0)	0 (0)	1.000	0 (0)	1 (2.9)	0.314
AJCC stage, I–II	10 (55.6)	12 (34.3)	0.137	10 (55.6)	11 (55.0)	0.973
Antiviral therapy	15 (42.8)	16 (45.7)	0.781	15 (42.8)	20 (57.1)	0.329
Recurrence	9 (25.7)	11 (31.4)	0.597	9 (25.7)	4 (11.4)	0.124
Mortality	14 (40.0)	16 (45.7)	0.629	14 (40.0)	20 (57.1)	0.151
Follow up times (months)	31 (4–75)	52 (1–98)	0.001	31 (4–75)	24 (1–85)	0.129

Data are presented as the median (range) or number (percentage)

*HBV* Hepatitis B virus, *HCV* hepatitis C virus, *INR* international normalized ratio, *AFP* alpha-fetoprotein, *ICG* indocyanine green, *AJCC* American Joint Committee on Cancer

**Table 3:** Comparison of hepatocellular carcinoma versus cholangiocarcinoma after propensity score matching

Variable	HCC (n = 43)	CC (n = 51)	*p* value
Male	28 (65.1)	33 (64.7)	0.213
Age (years)	59 (35–81)	60 (35–80)	0.967
Smoking	13 (30.2)	8 (15.7)	0.092
Alcohol use	11 (25.6)	4 (7.8)	0.099
HBV positive	21 (48.8)	26 (51.0)	0.836
HCV positive	9 (20.9)	18 (35.3)	0.125
Total bilirubin	0.85 ± 0.33	0.94 ± 0.46	0.241
Albumin	4.1 ± 0.4	4.2 ± 0.3	0.498
INR	1.05 ± 0.06	1.05 ± 0.12	0.912
Cirrhosis	3 (7.0)	6 (11.8)	0.432
Child–Pugh class A	39 (90.7)	51 (100)	0.058
Edmondson–Steiner Grades, I–II	5 (11.6)	12 (23.5)	0.379
Tumor size	5.7 ± 3.6	5.3 ± 2.4	0.596
Tumor size ≥ 5 cm	19 (44.4)	23 (52.3)	0.450
Tumor number (≥ 2)	1 (2.3)	2 (4.5)	0.570
AFP (ng/mL) ≥ 200	8 (18.6)	3 (5.9)	0.061
ICG%	9.2 ± 4.7	9.6 ± 5.5	0.686
Operative margin > 1 cm	24 (55.8)	30 (58.8)	0.749
Major hepatectomy	23 (53.4)	35 (68.6)	0.258
Microvascular invasion	9 (20.9)	8 (15.7)	0.510
Macrovascular invasion	6 (13.9)	1 (1.9)	0.057
Lympho nodules metastasis	2 (4.6)	8 (15.7)	0.062
Distal metastasis	0 (0)	3 (5.9)	0.106
AJCC stage, I–II	23 (60.4)	36 (70.5)	0.163
Antiviral therapy	18 (41.8)	25 (49.0)	0.186
Recurrence	16 (37.2)	6 (11.8)	0.004
Mortality	13 (30.2)	27 (52.9)	0.027
Follow up times (months)	61 (1–98)	26 (1–85)	< 0.0001

Data are presented as the median (range) or number (percentage)

*HBV* Hepatitis B virus, *HCV* hepatitis C virus, *INR* international normalized ratio, *AFP* alpha-fetoprotein, *ICG* indocyanine green, *AJCC* American Joint Committee on Cancer

**Fig. 3:**
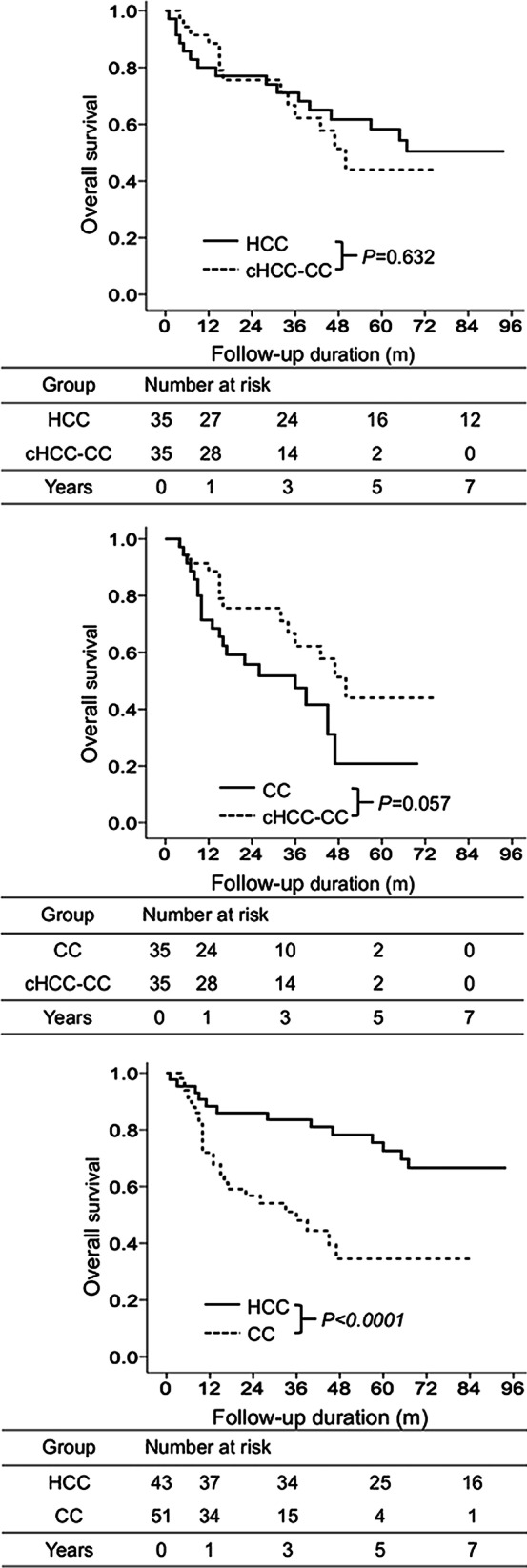
Overall survival of different groups after propensity score matching. Comparison of overall survival between cHCC-CC and HCC groups (**a**). Comparison of overall survival between cHCC-CC and CC groups (**b**). Comparison of overall survival between HCC and CC groups (**c**)

Comparing cHCC-CC and HCC groups after PSM (Table 2), there were 35 patients each in cHCC-CC and HCC groups. OS was not significantly different between cHCC-CC and HCC groups (*p* = 0.632, Fig. 3a). Cumulative OS rates at 1, 3, 5, and 7 years in cHCC-CC and HCC groups were 80.0%, 71.1%, 58.2%, and 50.4% and 88.5%, 62.2%, 44%, and 44.0%, respectively (Fig. 3a).

Comparing cHCC-CC and CC groups after PSM (Table 2), there were 35 and 35 patients in cHCC-CC and CC groups, respectively. OS was not significantly different between cHCC-CC and CC groups (*p* = 0.057, Fig. 3b). Cumulative OS rates at 1, 3, 5, and 7 years in cHCC-CC and CC groups were 88.5%, 62.2%, 44.0%, and 44.0% and 71.4%, 47.5%, 20.8%, and 20.8%, respectively (Fig. 3b).

Comparing HCC and CC groups after PSM (Table 3), there were 43 and 51 patients in HCC and CC groups, respectively. OS was significantly better in the HCC group than in the CC group (HR: 3.29, 95% CI: 1.62–6.64, *p* < 0.0001, Fig. 3c). Cumulative OS rates at 1, 3, 5, and 7 years in HCC and CC groups were 88.3%, 83.5%, 72.6%, and 66.6% and 72.0%, 48.1%, 34.5%, and 34.5%, respectively (Fig. 3c).

### Recurrence in different groups after PSM

**Fig. 4:**
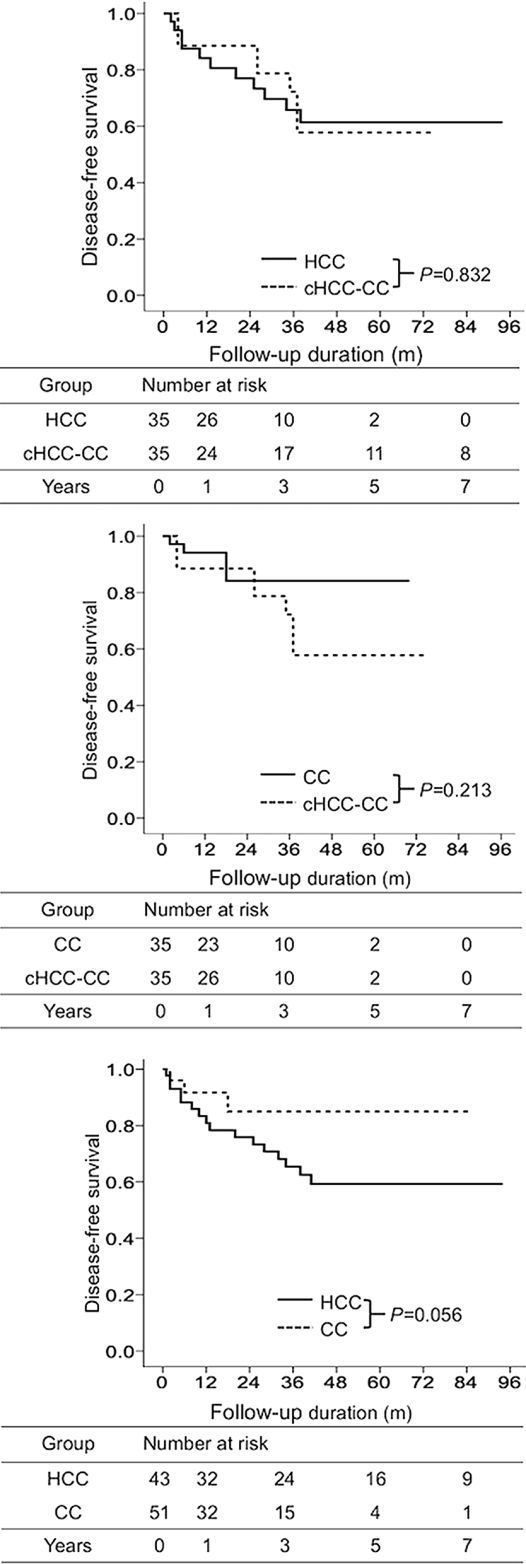
Disease-free survival in different groups after propensity score matching. Comparison of the disease-free survival between cHCC-CC and HCC groups (**a**). Comparison of the disease-free survival between cHCC-CC and CC groups (**b**). Comparison of the disease-free survival between HCC and CC groups (**c**)

Upon comparing cHCC-CC and HCC groups after PSM (Table 2), no significant intergroup difference was observed regarding the recurrence rate (*p* = 0.831, Fig. 4a). Cumulative DFS at 1, 3, 5, and 7 years in cHCC-CC and HCC groups were 88.6%, 72.2%, 57.7%, and 57.7% and 84.2%, 65.8%, 61.4%, and 61.4%, respectively (Fig. 4a).

When cHCC-CC and CC groups were compared after PSM (Table 2), no significant intergroup difference was noted regarding the recurrence rate (*p* = 0.213, Fig. 4b). Cumulative DFS at 1, 3, 5, and 7 years in cHCC-CC and CC groups were 88.6%, 72.2%, 57.7%, and 57.7% and 94.1%, 84.2%, 84.2%, and 84.2%, respectively (Fig. 4b).

Upon comparing HCC and CC groups after PSM (Table 2), no significant intergroup difference was observed regarding OS (*p* = 0.056, Fig. 4c). Cumulative DFS at 1, 3, 5, and 7 years in HCC and CC groups were 80.9%, 65.4%, 59.3%, and 59.3% and 91.7%, 84.9%, 84.9%, and 84.9%, respectively (Fig. 4c).

## Discussion

cHCC-CC is a rare type of PLC [1, 2], and its incidence accounts for 0.4–14.2% of PLC [1–5]. Our study results indicated that there were 35 (7.0%) patients with cHCC-CC out of the 505 patients who underwent surgery for PLC. The clinicopathological features of cHCC-CC are more identical to those of HCC than CC. The OS rate was significantly lower in the cHCC-CC group than in the HCC group. The OS rate was not significantly higher in the cHCC-CC group than in the CC group. After PSM, no significant differences were noted regarding the OS rate between the cHCC-CC group and the HCC or CC group. However, the OS rate was significantly higher in the HCC group than in the CC group before and after PSM. In addition, no significant differences were noted in terms of the DFS among cHCC-CC, HCC, and CC groups before and after PSM.

The clinicopathological features of the cHCC-CC group resembled those of the HCC group more than the CC group. Upon comparing cHCC-CC and HCC groups, no significant differences were observed regarding most demographic features, comorbidity, laboratory data, surgical methods, pathological characteristics, and tumor factors (including Edmondson–Steiner Grades I–II, tumor size, tumor number, microvascular invasion, macrovascular invasion, lymph node metastasis, AJCC stage I–II, and tumor recurrence) except for factors such as age, diabetes mellitus, tumor size ≥ 5 cm, lymph node metastasis, mortality, and mortality per 100 person-years. When cHCC-CC and CC groups were compared, significant differences were noted regarding diabetes mellitus, alcohol use, cirrhosis, AFP level ≥ 200 ng/mL, operative margin > 1 cm, major hepatectomy, microvascular invasion, and macrovascular invasion. Our study’s observation that the clinicopathologic features of cHCC-CC resembled those of the HCC group more than the CC group is inconsistent with the results of previous studies [7–12, 14].

Our study determined that median OS was 50.1 months in the cHCC-CC group after surgical resection. This median OS was higher than that reported in previous studies, which concluded that the median OS of patients with cHCC-CC ranged from 20 to 47 months [3, 5, 7, 10, 12, 15–21, 24, 25]. Nevertheless, our study’s finding of significantly lower OS in the cHCC-CC group before PSM is consistent with the results of previous studies [2, 5]. Notably, OS in the cHCC-CC group was not significantly different compared with the HCC or CC group after PSM. This result differs from those of previous studies after the stage-matched analysis [2, 5, 14]. We are the first to present the fact in the literature that no significant differences related to OS were observed between cHCC-CC and HCC groups after PSM. This finding is probably because the clinicopathologic features of cHCC-CC are similar to those of HCC, especially those related to tumor factors and tumor recurrence. Therefore, identical recurrence rates could have resulted in similar OS in cHCC-CC and HCC groups.

Our study revealed that the recurrence rate was not significantly different among cHCC-CC, HCC, and CC groups before and after PSM. Our results are consistent with those of previous studies that revealed that the DFS was not significantly different among cHCC-CC, HCC, and CC groups [2, 5, 14]. This finding is probably because of the similarity in the clinicopathologic features of cHCC-CC and HCC groups, especially regarding tumor factors, such as Edmondson–Steiner grades I–II, tumor size, tumor number, microvascular invasion, macrovascular invasion, lymph node metastasis, distal metastasis, AJCC stage I–II, and tumor recurrence.

The limitations of our study were the small sample size of cHCC-CC and CC groups. This small sample size could have resulted in statistically nonsignificant differences related to OS and recurrence after PSM. Second, we did not analyze the molecular markers, tumor markers, and immunohistochemical characteristics of patients with cHCC-CC.

## Conclusions

The clinicopathologic features of cHCC-CC resembled those of HCC more than CC. The OS rate was significantly lower in the cHCC-CC group than in the HCC group. The OS rate was not significantly different between cHCC-CC and CC groups. After PSM, the OS rate in the cHCC-CC group was not significantly different than that in the HCC or CC group. In addition, the DFS was not significantly different among cHCC-CC, HCC, and CC groups before and after PSM.

## Data Availability

Data is available from the corresponding author upon reasonable request.

## Abbreviations

cHCC-CC: Combined hepatocellular carcinoma and cholangiocarcinoma
HCC: Hepatocellular carcinoma
CC: Cholangiocarcinoma
PSM: Propensity score matching
CP class: Child–Pugh class
AJCC: American Joint Committee on Cancer
INR: International normalized ratio
AFP: Alpha-fetoprotein
OS: Overall survival
DFS: Disease-free survival
CI: Confidence interval
HR: Hazard ratio
